# Microbial Electrochemically Assisted Treatment Wetlands: Current Flow Density as a Performance Indicator in Real-Scale Systems in Mediterranean and Northern European Locations

**DOI:** 10.3389/fmicb.2022.843135

**Published:** 2022-04-05

**Authors:** Lorena Peñacoba-Antona, Carlos Andres Ramirez-Vargas, Colin Wardman, Alessandro A. Carmona-Martinez, Abraham Esteve-Núñez, Diego Paredes, Hans Brix, Carlos Alberto Arias

**Affiliations:** ^1^IMDEA Water, Parque Científico Tecnológico, Universidad de Alcalá, Madrid, Spain; ^2^METfilter S.L., Seville, Spain; ^3^Department of Analytical Chemistry, Physical Chemistry and Chemical Engineering, University of Alcalá, Madrid, Spain; ^4^WATEC, Aarhus University, Aarhus, Denmark; ^5^Department of Biology—Aquatic Biology, Aarhus University, Aarhus, Denmark; ^6^Water and Sanitation Research Group (GIAS), Universidad Tecnológica de Pereira, Pereira, Colombia

**Keywords:** constructed wetlands (CWs), electric potential sensor, electroactive bacteria (EAB), microbial electrochemical snorkel, METland, real-scale

## Abstract

A METland is an innovative treatment wetland (TW) that relies on the stimulation of electroactive bacteria (EAB) to enhance the degradation of pollutants. The METland is designed in a short-circuit mode (in the absence of an external circuit) using an electroconductive bed capable of accepting electrons from the microbial metabolism of pollutants. Although METlands are proven to be highly efficient in removing organic pollutants, the study of *in situ* EAB activity in full-scale systems is a challenge due to the absence of a two-electrode configuration. For the first time, four independent full-scale METland systems were tested for the removal of organic pollutants and nutrients, establishing a correlation with the electroactive response generated by the presence of EAB. The removal efficiency of the systems was enhanced by plants and mixed oxic–anoxic conditions, with an average removal of 56 g of chemical oxygen demand (COD) m_bed material_^–3^ day^–1^ and 2 g of total nitrogen (TN) m_bed material_^–3^ day^–1^ for Ørby 2 (partially saturated system). The estimated electron current density (*J*) provides evidence of the presence of EAB and its relationship with the removal of organic matter. The tested METland systems reached the max. values of 188.14 mA m^–2^ (planted system; IMDEA 1), 223.84 mA m^–2^ (non-planted system; IMDEA 2), 125.96 mA m^–2^ (full saturated system; Ørby 1), and 123.01 mA m^–2^ (partially saturated system; Ørby 2). These electron flow values were remarkable for systems that were not designed for energy harvesting and unequivocally show how electrons circulate even in the absence of a two-electrode system. The relation between organic load rate (OLR) at the inlet and coulombic efficiency (CE; %) showed a decreasing trend, with values ranging from 8.8 to 53% (OLR from 2.0 to 16.4 g COD m^–2^ day^–1^) for IMDEA systems and from 0.8 to 2.5% (OLR from 41.9 to 45.6 g COD m^–2^ day^–1^) for Ørby systems. This pattern denotes that the treatment of complex mixtures such as real wastewater with high and variable OLR should not necessarily result in high CE values. METland technology was validated as an innovative and efficient solution for treating wastewater for decentralized locations.

## Introduction

The treatment wetland (TW; a.k.a. as constructed wetlands—CW) is an engineered and sustainable nature-based system for the treatment and pretreatment of wastewaters of different origins (Langergraber et al., 2019). These systems mimic and optimize the physical, chemical, and biological processes occurring in natural wetlands (Dotro et al., 2017). These interactions lead to the occurrence of different mechanisms for pollutant removal, such as precipitation, sedimentation, filtration, volatilization, adsorption, plant uptake, and microbial-driven degradation (Kadlec and Wallace, 2008). The removal efficiency of TWs is determined by their design, operative settings (loading rate, loading pattern, etc.), and environmental conditions inside the wetland bed (e.g., substrate type, pH, temperature, dissolved oxygen, and redox conditions) (Wu et al., 2015). The TW is considered a robust and cost-effective technology that offers low-effort operation and maintenance, hence, is being extensively used worldwide as a developed decentralized wastewater treatment solution (Brix et al., 2007; Vymazal, 2009; Langergraber and Masi, 2018). However, one of the limiting factors of TW implementation is the footprint required to reach the desired treatment targets, which is much larger when compared with other conventional wastewater treatment technologies (Dotro et al., 2017). Therefore, to minimize surface area requirements, the use of more conventional treatment solutions or intensified wetland-based systems must be considered (Langergraber et al., 2019).

In the last decade, a combination of TWs with electrobioremediation strategies has been developed aiming at the intensification of TWs (Wang et al., 2020). Electrobioremediation relies on the metabolic activity of electroactive bacteria (EAB) capable of exchanging electrons from metabolism with electroconductive materials (Aguirre-Sierra et al., 2016; Ramírez-Vargas et al., 2018). EAB have been identified in different environments including natural aquatic sediments, aerobic/anaerobic sludge from wastewater treatment facilities, and also in wastewater (Aguirre-Sierra et al., 2016; Ramírez-Vargas et al., 2020). In microbial electrochemically assisted TW systems, several EAB strains have been identified as able to grow as electroactive biofilms. These biofilms are composed mainly, but not exclusively, of microorganisms from *Desulfuromonas*, *Pseudomonas*, *Shewanella*, and *Geobacter* genera (Aguirre-Sierra et al., 2016; Ramírez-Vargas et al., 2019; Saba et al., 2019; Rotaru et al., 2021). TWs operating under water-saturated conditions are anaerobic along almost their entire depth profile with anoxic/aerobic conditions only occurring in the uppermost section of the system at the water–air interface (Aguirre et al., 2005). These conditions generate a natural redox profile that matches the gradient of ions and electrons reported in other microbial electrochemical technologies (MET) such as microbial fuel cells (MFC) and combined TW–MFC systems (Yadav et al., 2012; Doherty et al., 2015a; Ramírez-Vargas et al., 2018; Kabutey et al., 2019; Srivastava et al., 2020). Applications of TW–MFCs at laboratory scale have expanded from treating conventional pollutants (Oon et al., 2016) and nutrients (Wang et al., 2016), to more persistent compounds such as oil (Yang et al., 2016), pharmaceuticals, or heavy metals (Nitisoravut and Regmi, 2017). Such studies show satisfactory removal rates and energy yields at a laboratory scale, but there are technical challenges to scale up these technologies and reach energy harvesting comparable with other sustainable sources.

In contrast with TW–MFCs, a different design has been developed in the past years by using an electroconductive bed under short circuit (snorkel configuration). Such system’s only aim is to optimize wastewater treatment and is known as microbial electrochemically assisted TW, or the METland^®^. In METlands, EAB growth is stimulated by transferring electrons to an electroconductive material that acts as an unlimited acceptor, therefore maximizing organic pollutant oxidation (Esteve-Núñez, 2015). The METland operates as a microbial electrochemical snorkel (Erable et al., 2011) using a conductive material to connect anoxic (anode) and oxic zones (cathode). Aguirre-Sierra et al. (2016) tested the first laboratory-scale METland for the removal of organic pollutants and nitrogen from real urban wastewater. The flooded configuration showed removal rates of 91% for chemical oxygen demand (COD) and 96% for biochemical oxygen demand (BOD_5_) (HRT = 0.5 day), 97% for NH_4_–N, and 69% for total nitrogen (TN) (HRT = 3.5 days). Similar results were shown by Ramírez-Vargas et al. (2019), in terms of organic removal rates using mesocosm set-ups treating real wastewater at loading rates of approximately 60 g m^–2^ day^–1^ and reached removal efficiencies of 90% for COD, 88% for BOD, 46% for NH_4_–N, and 86% for PO_4_–P. Interestingly, METlands can also be operated under non-flooded downflow conditions. Unexpectedly, EAB like *Geobacter* were found to coexist with nitrifying microorganisms, a system under oxic conditions (Aguirre-Sierra et al., 2020). Most recently, Prado et al. (2020) reported removals of up to 95% for COD and 71% for TN with the integration of an artificial device, the e-sink, for consuming electrons. These results suggest that the METland system can enhance biodegradation rates, reducing the footprint of classical TWs by 10-fold (Peñacoba-Antona et al., 2021b).

Moreover, direct and accurate measurements of the bioelectrochemical reactions in a METland system have been a challenge. The lack of electrodes and external circuits did not allow for the monitoring of electrical current flowing between an anode and a cathode. However, the charge imbalance due to the activity of electroactive biofilms inside a METland system is similar to the electrochemical potential differences between anodic and cathodic regions that exist in certain environments, as in the biogeobattery model (Ptushenko, 2020). Such charge differences create electric potentials (EPs) that trigger ionic/electron fluxes that can be detected in electrolyte conductors (Nielsen and Risgaard-Petersen, 2015). To detect those fluxes, tailor-made EP sensors could be used (Damgaard et al., 2014). These devices can collect low current signals in highly conductive matrixes and are insensitive to redox-active compounds that can affect EP readings. The measurement of EPs in METlands has been previously reported at the mesocosm scale (Ramírez-Vargas et al., 2019; Prado et al., 2020).

Based on the hypothesis that high electron currents in an electroactive system are correlated with high removal rates of pollutants, the study aimed to test the removal of organic matter in real-scale METland systems (treating wastewaters of different natures and at different geographical locations) and the correlation with the presence of EAB through the recording of electron current density in the water column. The measure of EP and current density profiles will establish different vertical profile zones in the METland bed regarding their bioelectrochemical activity and, consequently, their electrobioremediation performance. Indeed, the tested electrochemical strategy constitutes a tool for *in situ* monitoring of the performance of this new type of TW.

## Materials and Methods

### Design and Construction of METland Systems

**FIGURE 1:**
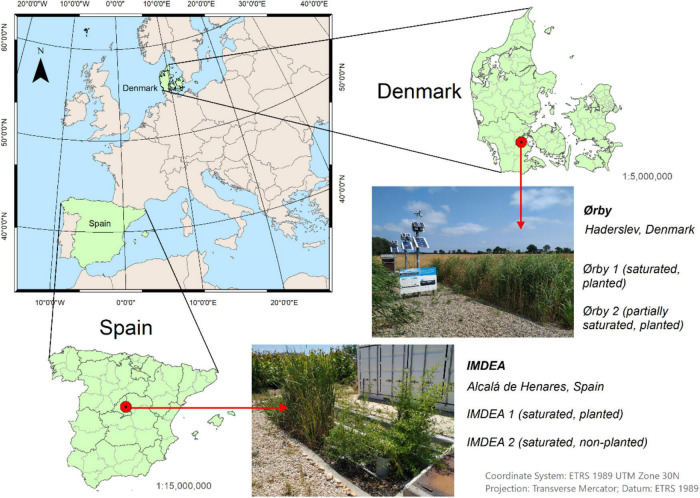
Location map of the METlands analyzed in the study.

The study was carried out in four full-scale METland systems constructed in two different latitudes: Mediterranean (Spain) and Northern Europe (Denmark) (Figure 1). Each location presented climatic and demographic conditions that allowed the testing of the technology under different environmental conditions. For instance, the climate in Southern Denmark is humid, with abundant and frequent precipitation throughout the year and cold winters. On the contrary, summers in the center of Spain are dry with scarce precipitation. Regarding the demographic distribution, decentralized households and small villages characterize southern Denmark. In each location, two METland beds were constructed to treat the local wastewater generated. Each METland system presents different configurations with plants adapted to the local climate. Regarding the wastewater flow, the systems were fed downflow and operated under saturated conditions. The material used as a substrate is an electroconductive coke (ec-coke) with the following characteristics: porosity 48% ± 1%, specific weight 0.8 ± 0.5 g ml^–1^, granulometry 0.3–1.0 ± 1.0 cm, and resistance 1.5 ± 0.5 Ω.

#### METland Unit at IMDEA Water (Spain)

**FIGURE 2:**
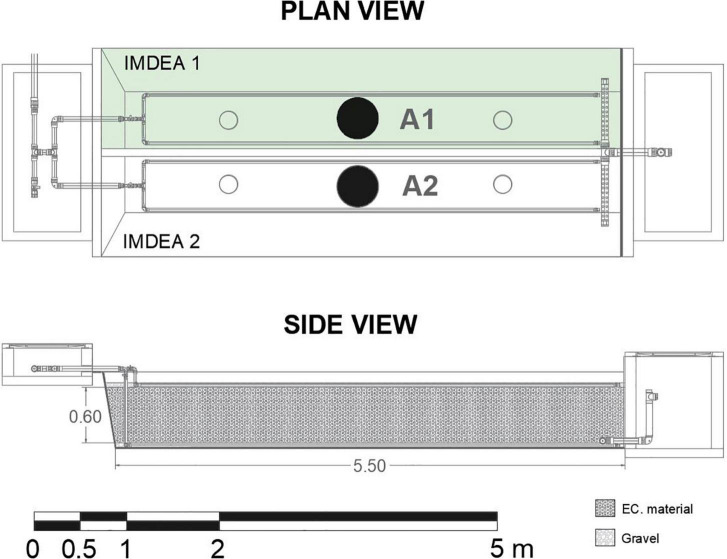
Plan view and profile of the METland system at IMDEA water (Alcalá de Henares). Sample points: A1 = planted with *Bambusa bambos*, *Typha angustifolia*, and *Iris germanica*; A2 = not planted.

The first treatment system was in the facilities of IMDEA Water (Alcalá de Henares, Spain). The system was built in January 2017 and has 5.5-m length, 2-m width, and 1.25-m depth (11 m^2^). The TW is divided into two separated chambers (5.5 m^2^ each), isolated with high-density polyethylene (HDPE) to avoid water fluxes to and from the beds (Figure 2). Each bed was filled with 0.6-m-deep electroconductive material and 0.05 m of gravel at the bottom, engulfing the drainage system built from Ø75 mm PVC perforated pipes. The two beds operated in parallel with a total effective surface area of 11 m^2^. The system operated as a vertical subsurface flow TW. The wastewater was distributed over the surface by Ø32-mm pressurized perforated pipes and flowed down through the filtering media. Treated water flowed to a chamber where the water level was controlled by a vertical pipe. The sampling points were in the middle of each bed (A1 and A2).

• IMDEA 1: with an effective surface area of 5.5 m^2^ and was planted with three different species, divided into three sections, from the influent to the effluent direction, *Bambusa bambos*, *Typha angustifolia*, and *Iris germanica.*
• IMDEA 2: with an effective surface area of 5.5 m^2^ without plants.

During the first year of operation, the system was fed with real urban wastewater generated at the research center in an intermittent flow regime varying from 0.5 to 2.0 m^3^ day^–1^. Solids from raw wastewater were removed by an Imhoff tank as a primary treatment. The influent water shows a low concentration of organic matter, as is expected from wastewater produced by an office building. The COD concentration was in a range of 50–130 mg l^–1^ and TN between 40 and 70 mg l^–1^ mainly in a reduced state (ammonium).

#### METland Unit at Ørby (Denmark)

**FIGURE 3:**
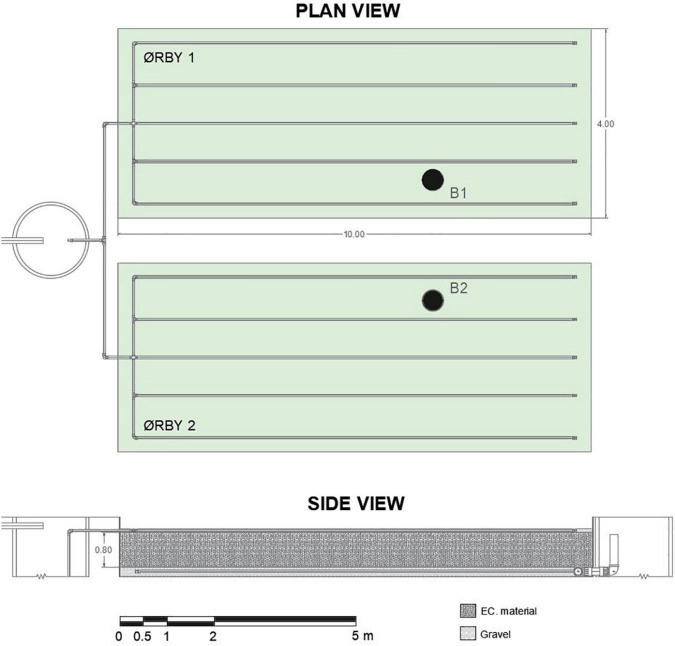
Plan view and profile of the METland system at Ørby. Both parallel systems were planted with *Phragmites australis*. Sample points B1 and B2 were located in the bed at the position marked in the figure.

A METland unit was built in the municipality of Ørby (Haderslev, Denmark) to treat domestic wastewater produced by a population equivalent (p.e.) of 200, with an effective surface area of 80 m^2^ in two beds each of 40 m^2^ (10 m × 4 m × 1 m deep). The beds were filled with 0.8-m ec-coke supplied by METfilter (Spain) (Figure 3). The wastewater was evenly distributed on the top of the beds. The perforated distribution pipes (PE Ø50 mm) were embedded in gravel to guarantee a homogeneous dispersal of the wastewater on the surface. Once wastewater was distributed on the surface, it flowed vertically down through the bed. Then, it was collected at the bottom by a Ø110-mm pipe manifold to evacuate it from the system to a chamber where the water level was regulated using swirling pipes.

• Ørby 1: a saturated bed with a surface area of 40 m^2^.
• Ørby 2: partially saturated bed (water level abated at 20 cm) with a surface area of 40 m^2^.

The urban community had a separate sewer system where no runoff was collected. Each of the ca. 40 houses was fitted with a septic tank as primary treatment, so pre-settled wastewater was transported to the treatment units by gravity. Water was collected in a pumping well that works as a homogenization tank before pulse feeding the METlands using a level-controlled pump. Each pulse delivered around 600 l that were evenly discharged on the surface of the beds at a rate of 300 l/pulse to each bed. The frequency of the pulses varied according to the water produced by the urban community, which varied by the day as well as throughout the year. The average wastewater characteristics were as follows: conductivity 1,600 μS cm^–1^, pH of 7.11, BOD_5_ 260–510 mg l^–1^, COD within the range of 540–910 mg l^–1^, total suspended solids (TSS) up to 100 mg l^–1^, and TN 60–110 mg l^–1^ mainly corresponding to ammonium. According to the data, the influent wastewater corresponds to an urban-type characterized by high COD and ammonium concentration (Metcalf and Eddy Inc, 2004).

### Sampling and Analysis of Pollutants (Physicochemical and Statistical)

Both systems were monitored for a period of 6 weeks, once a week, taking samples of the influent and the effluent for analyzing both organic matter (COD) and nutrients (PO4–P, TN, NH_4_–N, and NO_3_–N) to determine the removal performance of the systems. *In situ* measurements using calibrated electrodes and meters included pH (Hach PHC101), electrical conductivity (Hach sensION + 5060), temperature, dissolved oxygen (Hach LDO101), and redox potential (Hach MTC101) in the Ørby system. COD analysis was done by photometric evaluation (Hach LCI 400 cuvette test + DR 3900 spectrophotometer); TN was analyzed by combustion catalytic oxidation/NDRI method (Shimadzu TOC-VCPH); whereas orthophosphate (PO_4_–P), ammonia (NH_4_–N), and nitrate (NO_3_–N) were determined by ion chromatography (Lachat QuickChem^®^ 8000). All samples were analyzed following standard methods (APHA, 2012).

The removal efficiency (*E*) of the systems was evaluated with water measurements and mass balances at inlets and outlets according to Equation 1 (without considering the impact of evapotranspiration, where *V*_*in*_ and *V*_*out*_ correspond to the water inlet and outlet volume, respectively, and *C*_*in*_ and *C*_*out*_ correspond to the inlet and outlet concentrations of the monitored pollutants, respectively). Statistical analysis was conducted using the OriginPro 2019 statistical software. Thus, a one-way analysis of variance (ANOVA) was conducted to test the data’s statistical significance. The comparison among means was tested with Tukey’s test with a significance level of *p* < 0.05 (95% confidence).


(1) $$E=\frac{C_{\text{in}}-C_{\text{out}}}{C_{\text{in}}\times V_{\text{in}}}\times 100\%$$

### Microbial Electrochemical Activity

The evaluation of the microbial electrochemical activity of the full-scale METland systems was carried out based on the measurements of the EPs, estimation of ionic current densities (*J*), coulombic efficiencies (CE), and electron transfer rates. To measure the EP (mV), custom-made sensors, based on the design proposed by Damgaard et al. (2014), were used (*h*: 60 cm; Ø: 0.12 cm). The sensors were inserted in two different measuring ports in each METland. EP was measured at 1-cm intervals along the depth of the bed, with a resolution of ± 45 s, as previously reported (Ramírez-Vargas et al., 2019; Prado et al., 2020). To ease the graphical representation, the EP values (mV) were normalized using, as reference electrode, the water/atmosphere interface (0 mV at 0-cm depth). The ionic current density was calculated with an adapted version of Ohm’s Law (Equation 2) (Nielsen and Risgaard-Petersen, 2015), where *J* is the ionic current density (A m^–2^), σ is the electrical conductivity of water in the ports (S m^–1^), and *dΨ*/*dz* is the EP gradient (V m^–1^),


(2) J=-σ × dψ/dz×1/F

The CE, defined as the fraction of electrons recovered as current with regard to the maximum possible recovery from a substrate, was calculated based on Equation 3 (Logan et al., 2006). On Equation 3, *M* is the molecular weight of oxygen (32 g mol^–1^ O_2_), *I* is the current density (A m^–2^), *F* is the Faraday’s constant (96,485 C mol^–1^), *b* is the number of electrons exchanged per mole of oxygen (4 mol mol^–1^ O_2_), *q* is the hydraulic load rate (l m^–2^ s^–1^), and ΔCOD is the difference between influent and effluent concentrations of the substrate (g COD l^–1^),


(3) $$CE\left(\%\right)=\frac{MI}{Fbq△COD}\times 100$$

The electron transfer was estimated with an adapted version of the model presented by Risgaard-Petersen et al. (2014) (Equation 4), where *R*_*agg*_ is the aggregated electron transfer (μmol l^–1^ day^–1^) from anodic/cathodic reactions, *dJ/dz* is the gradient of current density between different levels inside the system (A m^–3^), and *F* is the Faraday’s constant (96,485 C mol^–1^),


(4) $$R_{agg}=-dJ/dz\times 1/F$$

## Results and Discussion

Full-scale METlands implemented at Mediterranean and Northern European locations using identical electroconductive material were tested and validated regarding (i) bioremediation performance and (ii) microbial electrochemical behavior. The following results revealed that such a variety of TWs is a promising configuration for treating urban wastewater of different natures.

### Treatment Performance

**FIGURE 4:**
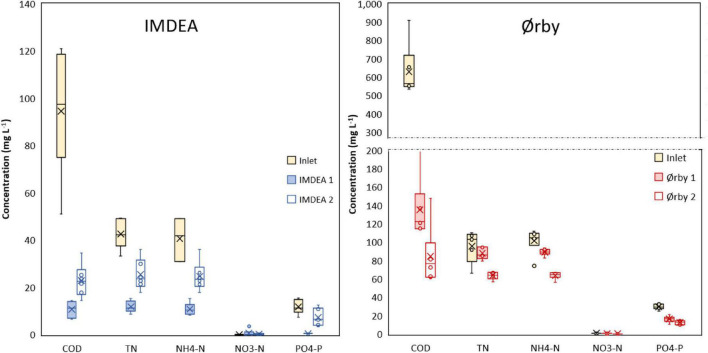
Concentrations of COD and nutrients from both inlet and outlet for METland units operating at IMDEA (Spain) and Ørby (Denmark). Within each box, a horizontal central line denotes median values; boxes extend from the 25th to the 75th percentile of each dataset; vertical lines denote adjacent values, the minimum and the maximum; and outliers are shown as circles.

The METlands constructed at Mediterranean (IMDEA) and Northern European (Ørby) locations were operated with real urban wastewater after primary treatment. In the case of IMDEA, the organic load of wastewater from an office building was lower than typical urban wastewater. In the case of Ørby, the organic load from urban wastewater was higher than at IMDEA, but due to seasonal variation, the flow rate was limited to 2 m^3^/day. Both situations revealed organic removal rates lower than other METland studies reported in the literature (Aguirre-Sierra et al., 2016; Prado et al., 2019; Ramírez-Vargas et al., 2019; Peñacoba-Antona et al., 2021b). Each system was analyzed independently considering the removal efficiencies based on inlet/outlet concentrations (Figure 4).

#### Case Study for Treating Wastewater From an Office Building (IMDEA Water) at a Mediterranean Location

At IMDEA, the systems reached average removal rates above 10.0 g of COD m_bed material_^–3^ day^–1^ (80%), 3.0 g of TN m_bed material_^–3^ day^–1^ (50%), 2.9 g of NH_4_—N m_bed material_^–3^ day^–1^ (47%), and 1.0 g of PO_4_–P m_bed material_^–3^ day^–1^ (61%) (Figure 4). COD removal efficiencies had slightly higher values in the planted section (IMDEA 1) compared to the non-planted section (IMDEA 2), with significant differences between planted and non-planted systems in terms of nitrogen removal. For the IMDEA units, the average COD removal was 10% higher in the planted system (IMDEA 1) in comparison to the non-planted (IMDEA 2), with significant differences between both parallel beds (*p* < 0.05). The differences might be related to the higher oxygen transfer through plant roots as previously reported in standard TWs (Brix, 1997; Saz et al., 2018). The results suggest a similar performance of the METlands regardless of the different water levels (see Supplementary Figure 1) or influent concentrations, due to the adaptability of the microbial community to different oxygen availabilities without impact on the efficiency for removing organic pollutants. COD removal efficiency was similar to the ones previously reported using METlands at the mesocosm scale (Aguirre-Sierra et al., 2016; Ramírez-Vargas et al., 2018).

The removal of nitrogen is performed in two stages: the first, nitrification, is typically promoted by aerobic conditions, and the second, denitrification, under anoxic conditions. Therefore, the combination of both conditions in the same system may increase the removal of TN (Cabred et al., 2019). TN removal from both IMDEA configurations showed significant statistical differences (*p* < 0.05). At IMDEA 1, plants enhanced the degradation of nutrients, reaching an average removal of 4.1 g of TN m_bed material_^–3^ day^–1^ (69%) in an anoxic system, compared to the removal of 2.0 g of TN m_bed material_^–3^ day^–1^ (35%) in the non-planted system (IMDEA 2). This improvement suggests the positive impact of vegetation in terms of ammonia removal, as previously reported in conventional TWs (Brix, 1997; Vymazal, 2010). Additionally, the oxygen supplied by the roots promotes aerobic conditions in the upper part of the system, enhancing the nitrification processes (oxidation of ammonia to nitrate), transforming 72% of NH_4_–N to nitrate (Aguirre et al., 2005). In addition, the anaerobic conditions under the water level promoted denitrification, achieving concentrations below the detection limit of nitrate in the effluent. In contrast, IMDEA 2 showed just a removal of 2.0 g of TN m_bed material_^–3^ day^–1^ (35%), suggesting a lower oxygen availability to transform ammonia into nitrate. These results were consistent with the 37% TN removal reported in a down-flow non-planted mesocosm METland (Aguirre-Sierra et al., 2016).

In terms of phosphorous removal, the IMDEA 1 system (planted bed) showed a maximum removal of 3.9 g of PO_4_–P m_bed material_^–3^ day^–1^ during the initial plant growth, surpassing the removal rates of the other METland systems analyzed and the rates reported in the literature about TWs (Kadlec and Wallace, 2008). After this initial period, the phosphorus removal stabilized at lower rates. Usually, the removal of phosphorus involves physical processes like precipitation or sorption, and in electroconductive materials, the PO_4_–P removal could be related to surface chemistry. Indeed, the chemistry of the electroconductive material is more complex than that of gravel and may have some metal content that favors the P adsorption (Prado et al., 2019). Additionally, these results suggest that the plants could enhance the phosphorus removal through uptake mechanisms as has been reported in TWs (Vymazal and Kröpfelová, 2008). On the other hand, IMDEA 2 accounts for an average removal of 40% of PO_4_–P (0.5 g PO_4_–P m_bed material_^–3^ day^–1^), presenting significant differences between planted and non-planted beds (*p* < 0.05).

#### Case Study for Treating Wastewater From an Urban Community (Ørby) at a North European Location

At the location in Denmark, the METland systems reached average removal rates above 51.3 g of COD m_bed material_^–3^ day^–1^ (80%), 2.1 g of TN m_bed material_^–3^ day^–1^ (20%), 2.6 g of NH_4_–N m_bed material_^–3^ day^–1^ (22%), and 1.6 g of PO_4_–P m_bed material_^–3^ day^–1^ (50%) (Figure 4). COD removal efficiencies had slightly higher values in the saturated section (Ørby 1) in comparison with the partially saturated section (Ørby 2). Thus, no significant differences were found between the two beds, with an average removal of 46.4 g of COD m_bed material_^–3^ day^–1^ (74%) in the saturated bed while 56.3 g of COD m_bed material_^–3^ day^–1^ (86%) in the partially saturated (Ørby 2), even though influent concentration was as high as 900 mg COD l^–1^, with an average organic load rate (OLR) of 52.0 g m^–2^ day^–1^. There were significant differences in TN removal, which was explained by the fact that the water level in one of the beds (Ørby 2) was abated at 0.20 m, favoring higher nitrification, and consequently, doubling the TN removal when compared to the saturated bed. The Ørby system had between 30 and 70% of PO_4_–P removal (0.8–2.4 g of PO_4_—P m_bed material_^–3^ day^–1^), without significant statistical difference regarding the water level between beds. These results suggest similar P removal rates to the ones achieved in METlands at mesocosm scale, that fluctuate between 40 and 76% of removal (Aguirre-Sierra et al., 2016; Ramírez-Vargas et al., 2019).

This study of full-scale METland systems corroborates the results obtained in the laboratory experiments (Aguirre-Sierra et al., 2016; Prado et al., 2019; Ramírez-Vargas et al., 2019). The results from the four different systems, together with the data from previous laboratory and mesocosms could set the bases for determining design parameters. Furthermore, it has demonstrated the viability of the technology, treating waters under different climatic conditions, water characteristics, and geographical locations (Peñacoba-Antona et al., 2021a).

### Bioelectrochemical Behavior of Full-Scale METlands

**FIGURE 5:**
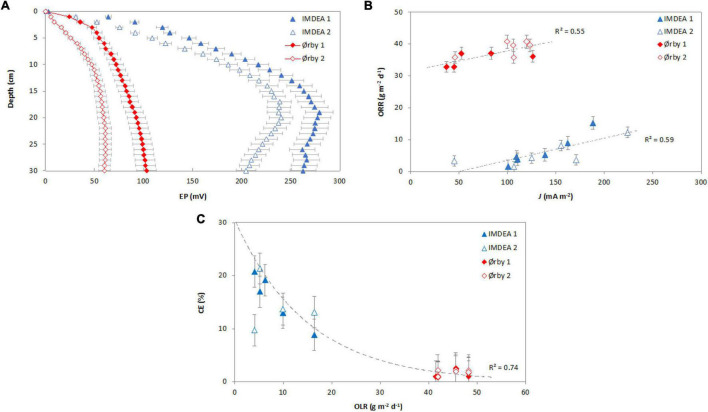
Bioelectrical response of tested METland systems. **(A)** Electric potential (EP) profiles along water depth; **(B)** the relation between electron flow density (*J*) and removed organic load rate (ORR) in terms of COD (%); **(C)** the relation between OLR at the inlet in terms of COD and coulombic efficiency—CE (%). In panel **(A)**, the EP profiles represent the average of different sampling values (for IMDEA systems: *n* = 18; for Ørby systems: *n* = 6). In panels **(B,C)**, each marker represents average values (for IMDEA systems: *n* = 3; for Ørby systems: *n* = 2). In all figures, error bars indicate the standard error of the mean.

The high efficiency of the METland for removing organic pollutants has been correlated with the metabolism of EAB through measuring the EP profiles at mesocosm scale (Ramírez-Vargas et al., 2019; Prado et al., 2020). These studies have revealed that EPs measured at different water depths typically shift if electron flow is taking place along the electroconductive bed. This variable was null in conventional TWs made of an inert material like gravel as reported elsewhere. The present study reports for the first time the EP profiles monitored at METland units operating at full scale under real conditions (Figure 5A). All METlands operated in the current study were made of identical electroconductive material, so, differences in the EP profile were due to the metabolic activity subjected to the different chemical compositions of the water and the operation of the system.

#### Electrochemical Behavior for Treating Urban Wastewater

At IMDEA 1 (planted), the electric field extended to a depth of ca. 19 cm with an electric potential of 278.93 mV, and in IMDEA 2 (non-planted) the electric field developed down to 20 cm with an electric potential of 239.90 mV. Likewise, for Ørby systems, the extension of electric fields along the water column was detected. For Ørby 1 (saturated), the electric field developed was 30 cm below the water level (the lowest level measured with the EP sensor) with an EP of 102.94 mV. Furthermore, in Ørby 2 (partially saturated), the electric field was developed to 27 cm below water level with an EP of 60.90 mV. These EP profiles were similar to those reported for METland-based mesocosm systems (Ramírez-Vargas et al., 2019; Prado et al., 2020), whose profiles showed the development of microbial electrical activity using different electroconductive materials (e.g. electroconductive coke and electroconductive biochar) as substrate media.

Besides the development of electric fields along the water depth in the systems, it was possible to identify differences in the maximal EP reached by each system. In the case of IMDEA systems, the highest EP was reached by the planted system (IMDEA 2); in the case of Ørby, the highest EP was reached by the system operating under partial saturation (Ørby 2). The highest potentials measured in the systems can be associated to the highest availability of O_2_ as the final electron acceptor. In the case of IMDEA 2 system, this could be associated with the oxygen released from the plants’ rhizomes together with diffusion from the atmosphere. In a conventional TW, the O_2_ availability due to the presence of roots promotes a gradient of oxidation-reduction potential between the upper and lower sections of the system (Aguirre et al., 2005); such oxygen presence enhances electron flows in METlands, and eventually, also removal rates (Prado et al., 2020). In the Ørby 2 system, a high oxygen availability can be generated by the oxygen availability inside the METland bed when it is fed intermittently, as has been reported in tidal flow TWs (Saeed et al., 2020) and mesoscale TW–MFC systems (Han et al., 2019).

Derived from the EP measured in the field, it was possible to estimate the electron current density (*J*) in the METland system, which provide evidence of the presence of EAB and its relationship with the removal of organic matter (Figure 5B). For IMDEA systems, the *J* values for the planted system (IMDEA 1) vary in the range of 100.58 to 188.14 mA m^–2^ and from 45.09 to 223.84 mA m^–2^ for the non-planted system (IMDEA 2). The difference between the planted and non-planted systems suggests a possible impact of plants on the microbial communities inside the METland system, facilitating the EAB metabolism, therefore boasting the removal of pollutants (Prado et al., 2022). Indeed, the oxygen exchange between the atmosphere and the plant root’s oxygen release increases the oxygen concentration in TWs (Brix, 1997), promoting higher oxidation-reduction potentials in the upper sections of the systems in contrast with the lower ones, therefore providing a redox gradient necessary for MET-based systems (Shen et al., 2018). In the case of the Ørby systems, the *J* values varied from 36.96 to 125.96 mA m^–2^ for the fully saturated system (Ørby 1) and between 45.84 and 123.01 mA m^–2^ for the partially saturated system (Ørby 2). Even though the system was not designed to harvest energy, the registered *J* values were comparable and even surpassed the current densities reported for TW–MET-based systems designed for simultaneous wastewater treatment and energy harvesting (Corbella et al., 2017; Ramírez-Vargas et al., 2018).

When assessing the association between electron current density (*J*) and the ORR, a positive relation between *J* and ORR was established (Figure 5B). However, despite the similarities in terms of bioelectrical response (expressed as *J*) between the systems, there were differences in terms of ORR, with higher values for Ørby systems in comparison to IMDEA ones. Such difference was probably due to the nature of the wastewater treated by every system (Figure 4), as well as differences in terms of OLR, with higher values for the Ørby systems in comparison to IMDEA systems. Low COD content (ca. 100 mg l^–1^) present in wastewater from office buildings may host a higher oxygen content capable of stimulating electron flow and eventually consuming the electrons generated by microbial oxidation. So, it is possible to use bioelectrical parameters such as electron current density (*J*) as an indicator of the removal of organic matter for METland systems. However, this kind of analysis should also consider other parameters like oxygen level or the presence of alternative oxidizing chemicals (e.g., nitrate).

Even though Ørby systems received a higher OLR in comparison to the IMDEA systems, the EPs showed an opposite pattern, with lower values in the Ørby systems and higher in IMDEA systems (Figure 5A). The result showed an impact on the bioelectrochemical productivity of the systems expressed in terms of CE. The relation between OLR and the CE of the systems showed a decreasing pattern (Figure 5C). In the case of systems at IMDEA, CE was between 8.8 and 53% (with OLR between 2.0 and 16.4 g of COD m^–2^ day^–1^), whereas in the Ørby systems, the CE values ranged from 0.8 to 2.5% (with OLR between 41.9 and 45.6 g of COD m^–2^ day^–1^). The CE values are within the reported ranges of the merging of TWs and other MET-based systems (Doherty et al., 2015a; Ramírez-Vargas et al., 2018).

The treatment of complex mixtures, such as real wastewater, with high and variable OLR should not necessarily result in high CE values. Indeed, the decrease of CE in MET-based systems as the OLR at influent increases has been previously reported (Ghangrekar and Shinde, 2008; Guadarrama-Pérez et al., 2019; Srivastava et al., 2020). The decrease can be attributed to different factors that may hinder the metabolic activity of electroactive microbial communities such as (i) the complexity of organic substrates in wastewater, (ii) the competition with abundant and varied microbial communities, (iii) the presence of anaerobic or methanogenic bacteria, (iv) the change of the internal electric resistance of conductive materials due to heterotrophic biofilm growth, (v) the physical removal of organic matter, and (vi) the increase in acidity affecting the growth of EAB and proton diffusion (Capodaglio et al., 2015; Doherty et al., 2015b; Corbella and Puigagut, 2018; Ramírez-Vargas et al., 2018; Hartl et al., 2019). Despite this decrease, the removal efficiencies from the METland systems were remarkable when operating under high OLR, and were a result of the interaction of different microorganism assemblages (Aguirre-Sierra et al., 2016; Ramírez-Vargas et al., 2020).

#### Impact on the Distribution of Anodic and Cathodic Zones

**FIGURE 6:**
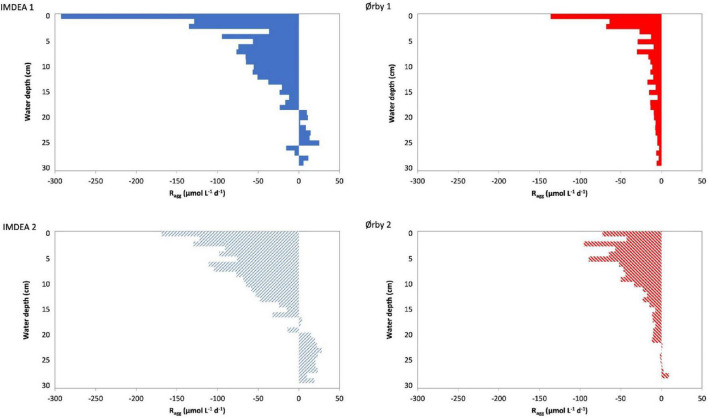
Aggregated electron transfer rate (*R*_*agg*_) for METland units treating wastewater (i) from an office building (planted IMDEA 1 and non-planted IMDEA 2) and (ii) from an urban community (fully saturated Ørby 1 and partially saturated Ørby 2). Positive values indicate zones where the bed is accepting electrons from an electron donor (anodic reaction), and negative values indicate zones where the bed is donating electrons (cathodic reactions).

Based on the local differences in electron fluxes, the electron transfer rates can be estimated (*R*_*agg*_) along the bed depth (Figure 6 and Supplementary Figure 2). Furthermore, such transfer rates allow for distinguishing anodic and cathodic zones, which match with the displacement of electric fields within the tested systems (Figure 5A). Thus, positive *R*_*agg*_ values represent the existence of an anodic zone, where the electrons are transferred from a donor (i.e., oxidation of organic pollutants) to an extracellular electron acceptor (electroconductive bed), and negative *R*_*agg*_ values represent a cathodic zone, where the electron transfer from the bed to bacteria is capable of reducing oxygen or nitrate (Prado et al., 2020).

In the case of the Ørby systems, operative conditions had an impact on the location and extension of both anodic and cathodic zones. In Ørby 1, there was a predominance of cathodic conditions that extended to 30 cm below the water level (*R*_*agg*_ of −5.85 μmol l^–1^ day^–1^), including a remarkable transfer rate reaching up to −136.27 μmol l^–1^ day^–1^ on the uppermost area of the system (Figure 6). This could be attributed to the saturated conditions, which limited the availability of terminal electron acceptors like O_2_ to the interphase in the uppermost zones, where exchange with the atmosphere or diffusion through the plant roots is possible. Additionally, NO_3_ can also play a role as an electron acceptor for denitrifying microorganisms. The lack of detectable NO_3_ in the effluent but the presence of ammonia in the influent shows strong evidence for nitrification (Figure 4). In Ørby 2, the cathodic zone reached 21 cm below the water level (*R*_*agg*_ of −11.89 μmol l^–1^ day^–1^), including a high cathodic activity present in the uppermost 10 cm (with *R*_*agg*_ between −95.43 and −42.83 μmol l^–1^ day^–1^). This distribution of the *R*_*agg*_ profile was mainly an effect of the partially saturated condition, where diffusion and mobilization of O_2_ from the atmosphere were promoted after feeding by a pulse; likewise, in Ørby 1, nitrate was not detected in the effluent.

Regarding IMDEA, the systems showed similarities between them in terms of the location of the cathodic and anodic zones, ca. 20 cm below the water surface (Figure 6). The main difference between them was the potential impact of the presence of plants in the IMDEA 1 system, which should contribute to higher O_2_ availability in the uppermost part of the bed. Likewise, in the Ørby systems, the presence of O_2_ and NO_3_ as a terminal electron acceptor contributes to the establishment of the cathodic and anodic zones, therefore allowing higher electron transfer values in the IMDEA 1 system (max. *R*_*agg*_ of −292.50 μmol l^–1^ day^–1^) than in the IMDEA 2 (max. *R*_*agg*_ of −168.70 μmol l^–1^ day^–1^). The cathodic and anodic zones detected in the systems seemed to be developed, not only by the type of configuration or operative conditions of the systems but also by the composition of the wastewater. The cathodic zones in the Ørby systems were deeper in comparison to the IMDEA systems, which could be associated with the highest OLR, whereas in the IMDEA systems, the electron transfer was higher than in the Ørby systems, a fact that could be derived from the relatively higher bioelectrochemical efficiency of the IMDEA systems that received a lower OLR. Likewise, in natural environments such as marine sediments or artificial electroactive biofilters like METlands, the assessment of electron fluxes is evident in the spatial mobilization of electrons from donors to acceptors that are physically in different environments (Prado et al., 2020). There are still open research questions and opportunities to study, in-depth, those dynamics of electron transfers that ultimately trigger an optimal performance of (EAB) in electrobioremediation systems.

## Conclusion

METlands operated at full scale are an innovative and effective solution for wastewater treatment, capable of reaching removal efficiencies of 90% COD (87 g of COD m_bed material_^–3^ day^–1^) and 70% TN (10.6 g of TN m_bed material_^–3^ day^–1^). This was clearly shown in these two case studies operating at different geographical locations with different wastewater compositions.

The study suggests the possibility of using bioelectrochemical parameters such as electron fluxes (*J*) to monitor the performance of a METland system in terms of organic matter removal. Keeping in mind that the correlation between electron fluxes and organic matter removal is site-specific, as a future perspective, these results open the possibility for using the current densities to monitor the performance remotely. In addition, the EP monitoring, the estimation of electron fluxes (*J*), and the electron transfer rate (*R*_*agg*_) calculations would allow for the detection of the most active zones inside the systems.

In summary, the bioelectrochemical behavior of full-scale electrobioremediation systems is not only a consequence of its operational conditions or configuration but is also affected by the type and composition of the influent wastewater. Lastly, METland technology was validated as an innovative and efficient solution for treating wastewater in decentralized locations.

## Data Availability Statement

The raw data supporting the conclusions of this article will be made available by the authors, without undue reservation.

## Author Contributions

CR-V, LP-A, CW, AC-M, DP, and CA: conceptualization. CR-V and LP-A: data curation, formal analysis, methodology, investigation, software, and visualization. AE-N, CA, and HB: funding acquisition and supervision. CR-V, LP-A, AE-N, and CA: writing—original draft. All authors contributed to the article and approved the submitted version.

## Conflict of Interest

The authors declare that the research was conducted in the absence of any commercial or financial relationships that could be construed as a potential conflict of interest.

## Publisher’s Note

All claims expressed in this article are solely those of the authors and do not necessarily represent those of their affiliated organizations, or those of the publisher, the editors and the reviewers. Any product that may be evaluated in this article, or claim that may be made by its manufacturer, is not guaranteed or endorsed by the publisher.
