# Supplementary material for: Microbial Electrochemically Assisted Treatment Wetlands: Current Flow Density as a Performance Indicator in Real-Scale Systems in Mediterranean and Northern European Locations
Source: Front Microbiol. 2022 Apr 5;13:843135. doi: 10.3389/fmicb.2022.843135 (PMC9016324; doi:10.3389/fmicb.2022.843135)
Supplement: Supplementary file 1 [file Data_Sheet_1.pdf]

Supplementary Material

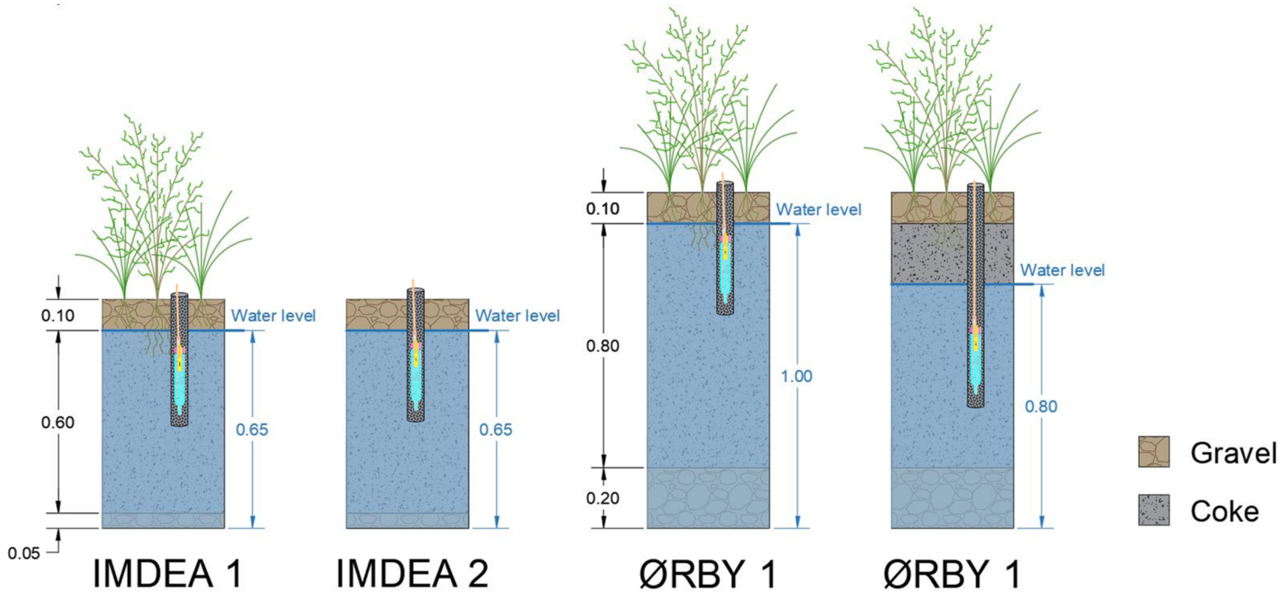

Supplementary Figure 1. Water level differences among tested METland systems.

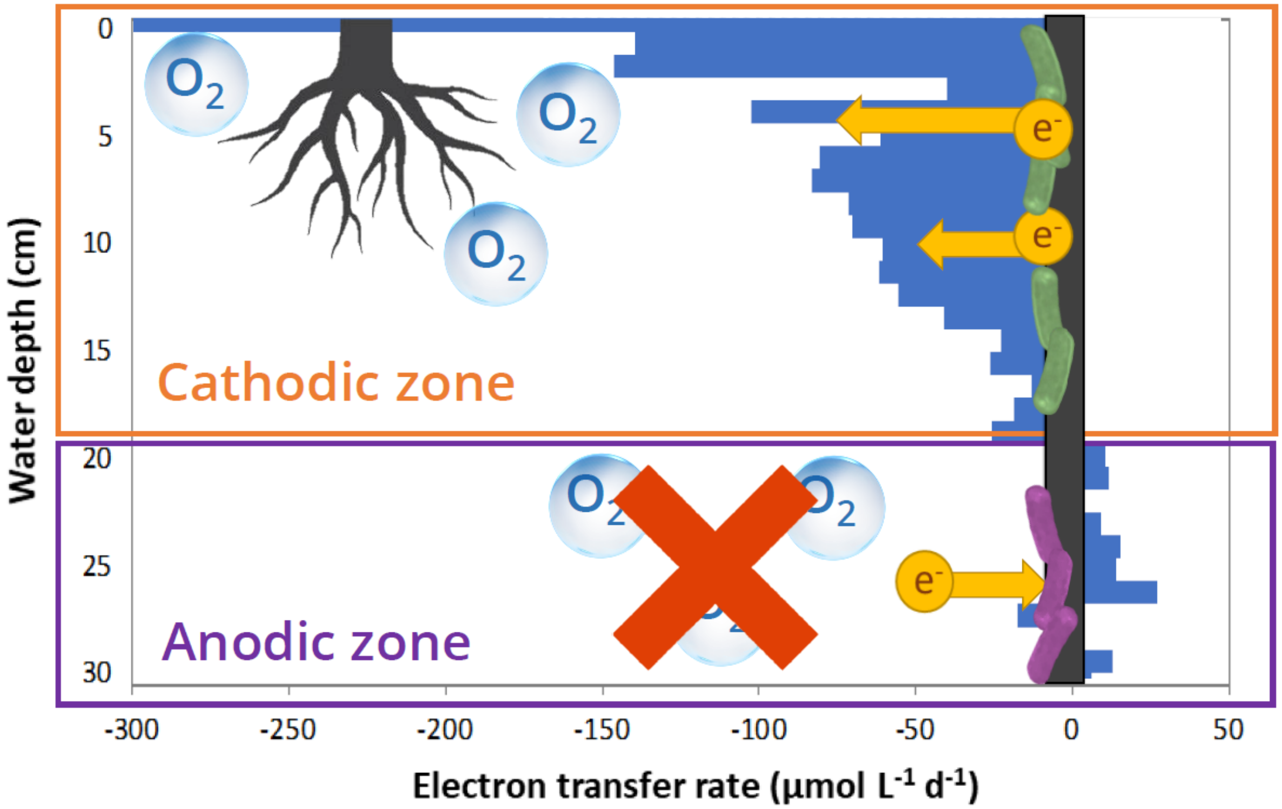

Supplementary Figure 2. Representation of cathodic and anodic zones as function of depth in tested METland systems.
